# Coronary calcification as a predictor of cardiovascular mortality in advanced chronic kidney disease: a prospective long-term follow-up study

**DOI:** 10.1186/s12882-019-1367-1

**Published:** 2019-05-28

**Authors:** Marta Cano-Megías, Pablo Guisado-Vasco, Hanane Bouarich, Gabriel de Arriba-de la Fuente, Patricia de Sequera-Ortiz, Concepción Álvarez-Sanz, Diego Rodríguez-Puyol

**Affiliations:** 1 ‘Principe de Asturias’ University Hospital, Ctra Alcalá-Meco s/n. Alcalá de Henares, 28805 Madrid, Spain; 2 European University, Internal Medicine, Ruber Juan Bravo Hospital, Juan Bravo St 39-49, ZP 28006 Madrid, Spain; 3 Guadalajara University Hospital, Donante de Sangre St s/n, ZP 19002 Guadalajara, Castilla La Mancha Spain; 4 ‘Infanta Leonor’ University Hospital, Gran vía del Este, 80, 28031 Madrid, Spain; 5 Research Foundation of ‘Principe de Asturias’ University Hospital, Ctra Alcalá-Meco s/n, Alcalá de Henares, 28805 Madrid, Spain

**Keywords:** Chronic kidney disease, Coronary artery calcification, Haemodialysis, Cardiovascular mortality, Coronary calcification score

## Abstract

**Background:**

Patients with advanced chronic kidney disease (CKD) exhibit higher prevalence of coronary artery calcification (CaC) than general population. CaC has been proposed as a risk factor for mortality in end-stage CKD, but most studies in the field are based on short-term follow-up.

**Methods:**

We conducted a cohort, 10-year prospective longitudinal study of consecutive cases referred to the renal unit. A non-enhanced multislice coronary computed tomography was performed at baseline. CaC was assessed by Agatston method. Patients were stratified according to their CaC score: severe calcification group (CaCs< 400 HU) and mild-moderate calcification group (CaCs≥400 HU). The overall and cardiovascular (CV) mortality, CV events, and factors potentially associated with CaC development were recorded.

**Results:**

137 patients with advanced CKD were enrolled and provided consent. Overall mortality rate was 58%; 40% due to CV events. The rate of overall mortality in the severe calcification group was 75%, and 30% in the low calcification group, whereas the rate of CV mortality was 35% vs. 6%, respectively (*p* < 0.001). The severe calcification group was older, had higher prevalence of type 2 diabetes mellitus, former cardiologic events, and lower albumin serum levels than the mild-moderate calcification group. In a multivariate Cox model, severe CaC was a significant predictor of CV mortality (HR 5.01; 95%CI 1.28 to 19.6, *p* = 0.02).

**Conclusions:**

Among advanced CKD, there was a significantly increase of CV mortality in patients with severe CaCs during a 10-year follow-up period. CaCs could be a useful prognostic tool to predict CV mortality risk in CKD patients.

**Electronic supplementary material:**

The online version of this article (10.1186/s12882-019-1367-1) contains supplementary material, which is available to authorized users.

## Background

Cardiovascular (CV) disease is one of the leading causes of death and premature mortality among chronic kidney disease (CKD) patients [1]. In addition to classic risk factors for CV morbimortality, different uraemia related conditions such as malnutrition, chronic inflammation, accelerated atherosclerosis and endothelial dysfunction have been considered to be involved in the genesis of CV damage [2, 3]. Additionally, bone and mineral derangements are well-recognized complications of CKD that might result in aberrances in bone turnover and calcification of vascular or other soft tissues [4, 5].

CV *calcifications affect most* of *CKD patients (60–90%)* and these calcifications develop *early* in the course of the *disease* [1, 6–9]*.* In haemodialysis, previous studies have suggested that coronary calcification (CaC) analysis could improve primary risk prediction of CV disease [1, 2, 10]. An elevated CaC score (CaCs> 400 HU) could be associated with higher overall and CV mortality [2, 10]. However, most of the studies have been performed after short follow-up, and scarce information is available concerning long-term prognosis [1]**.** Moreover, in advanced CKD and hemodialysis patients, the medial arterial wall calcification might predominate over atherosclerotic damage, and therefore it could be no underlying coronary obstructive atherosclerosis. Some studies have found poor correlation between CaCs measured by MSCT and coronary stenosis detected by coronary angiography [11–14]. Consequently, in these patients may predominate the non-atheromatous cardiovascular events [14]. Therefore, it is necessary to define the predictive role of CaCs for CV mortality in this population.

The primary aim of our study is to test, in long-term follow-up, the predictive value of CaCs detected by multi-layer spiral computed tomography (MSCT) for overall and CV mortality in advanced CKD patients, including those on hemodialysis. Secondary aims include assessing the prevalence of CaC and its effect on hospitalization due to CV events. As well as evaluating the potential clinical and biochemical factors involved in the development of severe CaC.

## Methods

We conducted a prospective, observational and 10-year follow-up study. All patients evaluated at the Renal Unit of ‘Principe de Asturias’ University Hospital during a 2-year period were considered for inclusion. A total of 137 patients with advanced CKD were enrolled. Inclusion criteria were as follows: age over 18 years, advance CKD disease (CKD stages 4-5) and patients on chronic haemodialysis (more than 6 months in the dialysis program). Exclusion criteria were as follows: acute kidney failure, pregnancy, a disease condition which could limit life expectancy to less than one year, and the inability to perform MSCT. Written informed consent was provided to all subjects. The Research Ethics Committee of the Hospital approved the study protocol.

As part of the initial evaluation in the renal unit, within one month prior to performing MSCT, in all cases a blood basal sample was collected for routine biochemistry analysis. All subjects underwent a synchronized MSCT to assess the basal CaC. A 16-detector MSCT Lightspeed Plus GE Medical System was used. Image processing was carried out in an Advantage Workstation 4.0 console. CaCs was calculated by adding the calcium score in the left main coronary artery, the left anterior descending, circumflex and right coronary artery. The CaCs described by Agatston [15] was calculated. It establishes a threshold of 130 Hounsfield Units (HU) to determine whether the lesions are calcified. The “Agatston score” incorporates the density of calcification, multiplying the calcification area by a weighted density coefficient. According to previously published information, patients may be classified into four groups according to baseline CaCs: no calcification (CaCs 0 HU); mild calcification (CaCs 1–100 HU); moderate calcification (CaCs 101–400 HU) and severe calcification (CaCs≥400 HU) [16–18]. Our patients were stratified into two groups in order to analyse the proposed outcomes: severe calcification group (CaCs≥400 HU) and mild-moderate calcification group (CaCs< 400 HU) [16].

Baseline clinical data were recorded from medical reports and are detailed in Table 1. The biochemical parameters included those related to renal function, bone mineral metabolism and classic cardiovascular risk variables. During the follow-up period, any new CV event that caused hospital admissions (heart failure, atrial fibrillation, stroke, ischemic heart disease) was recorded. For analysis purpose, we use a strategy of introducing a variable to the first event in time [17].

**Table 1:** Baseline patient characteristics and laboratory values

Variable	N (%)/Median (IQR)/Mean (SD)
Male (%) Female (%)	74 (54) 63 (46)
Age (years)	66 (52–71)
Haemodialysis (%)	85 (61.6)
CKD stage 4–5 (%)	52 (38.4)
Haemodialysis vintage (months)	25 (10.0–53)
Hypertension (%)	123 (89)
Diabetes mellitus (%)	38 (27.7)
Smoker (%)	30 (18.4)
Chronic ischemic heart disease (%)	27 (20.1)
Previous stroke event (%)	15 (11.3)
Previous cardiologic event (%)	35 (26.3)
Antiplatelet therapy (%)	48 (35.0)
Statin therapy (%)	59 (43.1)
Vitamin D therapy (%)	72 (53.3)
Calcium salt use (%)	73 (54.1)
Phosphate binder use (%)	57 (42.5)
Cinacalcet treatment (%)	9 (6.70)
CACI score (HU)	137
1 (0)	18 (13.1)
2 (1–100)	18 (13.1)
3 (101–400)	17 (12.4)
4 (≥ 400)	84 (61.3)
Cholesterol (mg/dl)	175 (±43.9)
LDL- cholesterol (mg/dl)	92.19 (±38.3)
HDL-cholesterol (mg/dl)	44.5 (±14.9)
Triglycerides (mg/dl)	142 (85–214)
Uric acid (mg/dl)	6.99 (± 1.54)
HbA1c (%)	6.56 (±1.62)
Albumin (g/dl)	4.05 (±0.44)
eGFR (ml/min/m^2^)	11.5 (4.8–22.7)
Creatinine (mg/dl)	7.51 (3.52–10.0)
Urea (mg/dl)	151 (±48.9)
Phosphorous (mg/dl)	4.65 (3.80–5.63)
Calcium (mg/dl)	9.38 (±0.72)
Calcium-phosphorus product	41.93 (36.1–50.7)
Bicarbonate (mmol/l)	19.63 (±4.99)
Intact PTH (pg/ml)	220 (94–413)
TSH (uIU/ml)	1.28 (0.79–2.78)
C-reactive protein (mg/l)	5.9 (3.13–13.1)

The overall mortality was defined as time from the recruitment moment to death from any cause, in-hospital or outpatient. CV mortality was considered as death due to myocardial infarction, congestive heart failure, cardiac arrhythmia, sudden cardiac death, peripheral vascular disease, or stroke.

### Statistical analysis

Quantitative results were expressed as mean ± standard deviation (SD) for continuous variables with normal distribution or as a median + interquartile range (IQR) in all other cases, in accordance with the Kolmogorov-Smirnov distribution test. Categorical variables are indicated as percentages related to the size of the sample. Subjects were split into two groups based on CaCs derived from baseline CT, CaCs ≥400 HU and CaCs< 400 HU. To compare both calcification groups, we used chi square test for binominal variables and Student t-test or Kruskal-Wallis test for quantitative variables, as suitable.

The influence of each variable on overall and CV mortality was assessed by means of a Cox proportional hazard models. The first stage consisted of a univariate analysis selecting those with a *p*-value < 0.1 as statistically significant. Then, a multivariate Cox approach was carried out, using a backward strategy (p-value < 0.05). The multivariate Cox regression model was used, assuming there is a maximum of one variable per each 10 events. Logistic regression analysis was used to identify the independent risk factors for high values of CaCs. Overall and CV mortality was estimated using the Kaplan-Meier (KM) method and Mantel-Cox (log-rank test) to compare survival curves. The omnibus method was used to adjust the chi-square output. We also took into consideration the strategy reported by Detrano et al. [18] to study how a doubling of the CaCs influenced on overall and CV mortality of our series. To transform CaCs into a continuous variable, the base-2 logarithm of the sum of the CaCs plus 1 (log_2_ [CaCs + 1]) was used. Each unit difference in the log-transformed CaCs represents a doubling of the score. Data were analysed using SPSS version 20.0 (SPSS Software, Chicago, IL, USA). The significance level was set at *p* < 0.05.

## Results

The population-based sample was composed of 137 patients, 85 subjects (62%) undergoing maintenance haemodialysis and 52 patients (38%) with advanced CKD. Subjects were 74 males and 63 females, with a median age of 66 years (IQR 52 to 71). Haemodialysis vintage was 25 months (IQR 10–53). The median follow-up period was 87.5 months (29.5–111). Regarding renal replacement therapy, at baseline, two out five of patients in HD were dialyzed with a membrane of high dialytic efficacy and in four out five it was used a standard dialysis bath. The main baseline subject characteristics are outlined in Table 1.

### Variables associated with high CaCs

The prevalence of CaC was 87% (range: 0 to 8798 HU). The median of CaCs was 600 HU (IQR 70–1794). Those patients with severe CaC (CaCs ≥400 HU) were older, had a longer haemodialysis vintage, showed higher prevalence of type 2 diabetes, previous cardiologic events, and exhibited higher triglyceride serum concentration and lower serum albumin levels than mild-moderate CaC subjects (CaCs< 400 HU) (Table 2.)

**Table 2:** Comparison between demographic, clinical and laboratory variables at study enrolment in patients with a high and a low coronary calcium score

Variable	CaCs ≥400 HU (*n* = 84)	CaCs < 400 HU (*n* = 53)	p
Age (years)	65.2 (±11.7)	56.3 (±14.9)	< 0.001*
Haemodialysis vintage (months)	56.2 (±65.1)	29.8 (±43.8)	0.025*
Haemodialysis (%)	71.1 (*n* = 59)	28.9 (*n* = 24)	0.004
Hypertension (%)	62 (*n* = 76)	38 (*n* = 46)	0.58
Diabetes mellitus (%)	83.8 (*n* = 32)	16.2 (*n* = 6)	< 0.001
Smoker (%)	66.7 (*n* = 20)	33.3 (*n* = 10)	0.10
Previous stroke event (%)	15 (*n* = 12)	5.8 (*n* = 3)	0.16
Previous cardiologic event (%)	88.2 (*n* = 31)	11.2 (*n* = 4)	< 0.001
Antiplatelet use (%)	81.2 (*n* = 39)	18.8 (*n* = 9)	< 0.001
Calcium salt use (%)	63.4 (*n* = 46)	36.6 (*n* = 26)	0.59
Phosphate binder use (%)	48.8 (*n* = 40)	31.4 (*n* = 15)	0.07
Vitamin D use (%)	64.8 (*n* = 47)	35.2 (*n* = 25)	0.37
Cholesterol (mg/dl)	170 (±39.6)	183 (±49.1)	0.085
LDL- cholesterol (mg/dl)	87.8 (±36.7)	101 (±40.2)	0.12
HDL-cholesterol (mg/dl)	43.1 (±11.1)	44.1 (±11.6)	0.72
Triglycerides (mg/dl)	177 (±107)	145 (±87.4)	0.01*
Uric acid (mg/dl)	6.97 (±1.59)	7.01 (±1.48)	0.89
HbA1c (%)	6.69 (±1.69)	6.17 (±1.41)	0.39
Albumin (g/dl)	3.94 (±0.41)	4.21 (±0.43)	0.001
eGFR (ml/min/m^2^)	12.3 (±9.65)	16.8 (±10.9)	0.045*
Creatinine (mg/dl)	7.58 (±3.75)	6.48 (±3.77)	0.01*
Urea (mg/dl)	152 (±48.9)	150 (±49.8)	0.75
Phosphorous (mg/dl)	4.87 (±1.32)	4.85 (±1.58)	0.39*
Calcium (mg/dl)	9.35 (±0.7)	9.41 (±0.75)	0.65
Calcium - phosphorus product	45.3 (±12.1)	45.6 (±15.4)	0.48*
Bicarbonate (mmol/l)	19.7 (±5.09)	19.3 (±4.83)	0.62
Intact PTH (pg./ml)	333 (±414)	324 (±326)	0.52*
TSH (uIU/ml)	4.52 (±10.9)	1.8 (±1.1)	0.48*
C-reactive protein (mg/l)	15.7 (±27.6)	12.8 (±23.4)	0.43*

*Kruskal-Wallis test, as appropriate

Variables that showed a significant association with severe CaCs (≥400 HU) were: age (OR 1.05; 95% confidence interval [CI] 1.02–1.08), haemodialysis (OR 2.85; 95%CI 1.40–5.83), type 2 diabetes (OR 4.92; 95%CI 1.89–12.8), previous cardiologic events (OR 7.59; 95%CI 2.49–23.2) and low serum albumin values (OR 2.92; 95%CI 1.31–6.50). On the multivariate regression analysis, age (OR 1.07; 95%CI 1.03–1.11), haemodialysis (OR 5.22; 95% CI 1.88–14.5) and type 2 diabetes (OR 6.21; 95%CI 1.81–21.3) kept a statistically significant association with severe CaCs.

### Mortality

During the follow-up period, 58% of subjects died and 40% of deaths were related to CV events. Considering overall mortality, 75% of patients (*n* = 60) were receiving haemodialysis and 25% (*n* = 20) had advanced CKD. In terms of CV mortality, 78% (*n* = 25) of subjects were on maintenance dialysis. Median overall survival, estimated by the inverted KM curve, was 44.6 ± 3.7 months (95% CI 37.4–51.8), while median CV survival was 39.1 ± 5.6 months (95% CI 28.1 to 50.1).

**Fig. 1:**
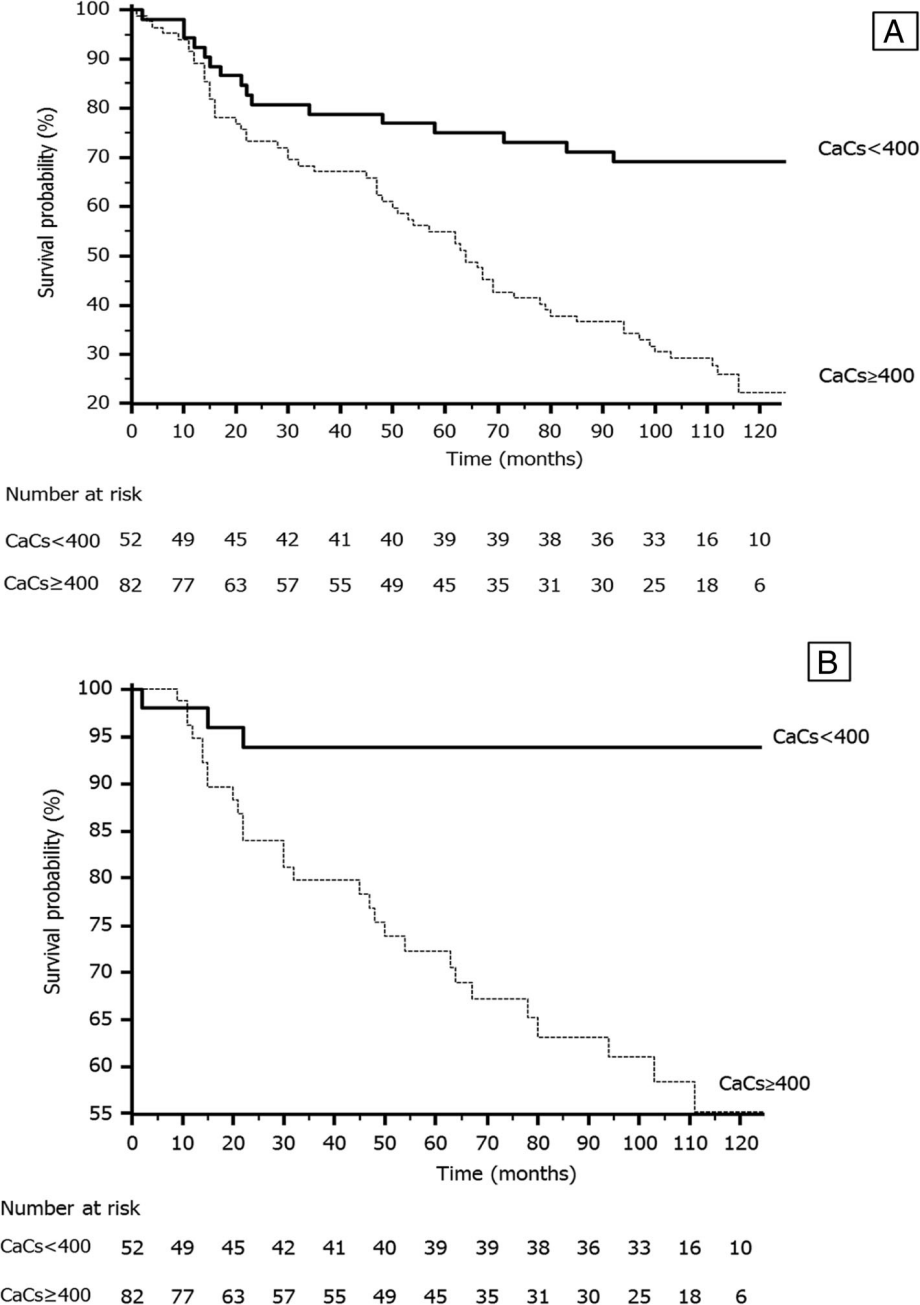
Unadjusted Kaplan Meier cumulative-curves for overall mortality (**a**) and cardiovascular mortality (**b**) according to coronary arterial calcification score in Hounsfield Units (CaCs HU): Group with severe coronary calcification (CaCs≥400 HU) and group with mild-moderate coronary calcification (CaCs< 400 HU) are shown *p* < 0.001

Overall mortality rate according to baseline CaCs was 17% (*n* = 3) in patients without CaC, 22% (*n* = 4) in patients with mild CaC, 53% (*n* = 9) in patients with moderate CaC and 75% (*n* = 64) in patients with severe CaC. Most of overall mortality events happened in group 4 (80%)**.** By KM analysis, patients with CaCs ≥400 HU had a significantly higher mortality compared to those with CaCs< 400 HU (X^2^ 18.92, *p* < 0.001) (Fig. 1a). There were no CV deaths in groups without or with mild CaC. Most of CV deaths belonged to the severe calcification group (91%, *n* = 29)**.** By KM analysis, patients with CaCs ≥400 HU showed a significantly higher CV mortality compared to those with CaCs< 400 HU (X^2^ 14.92, p < 0.001) too (Fig. 1b).

**Fig. 2:**
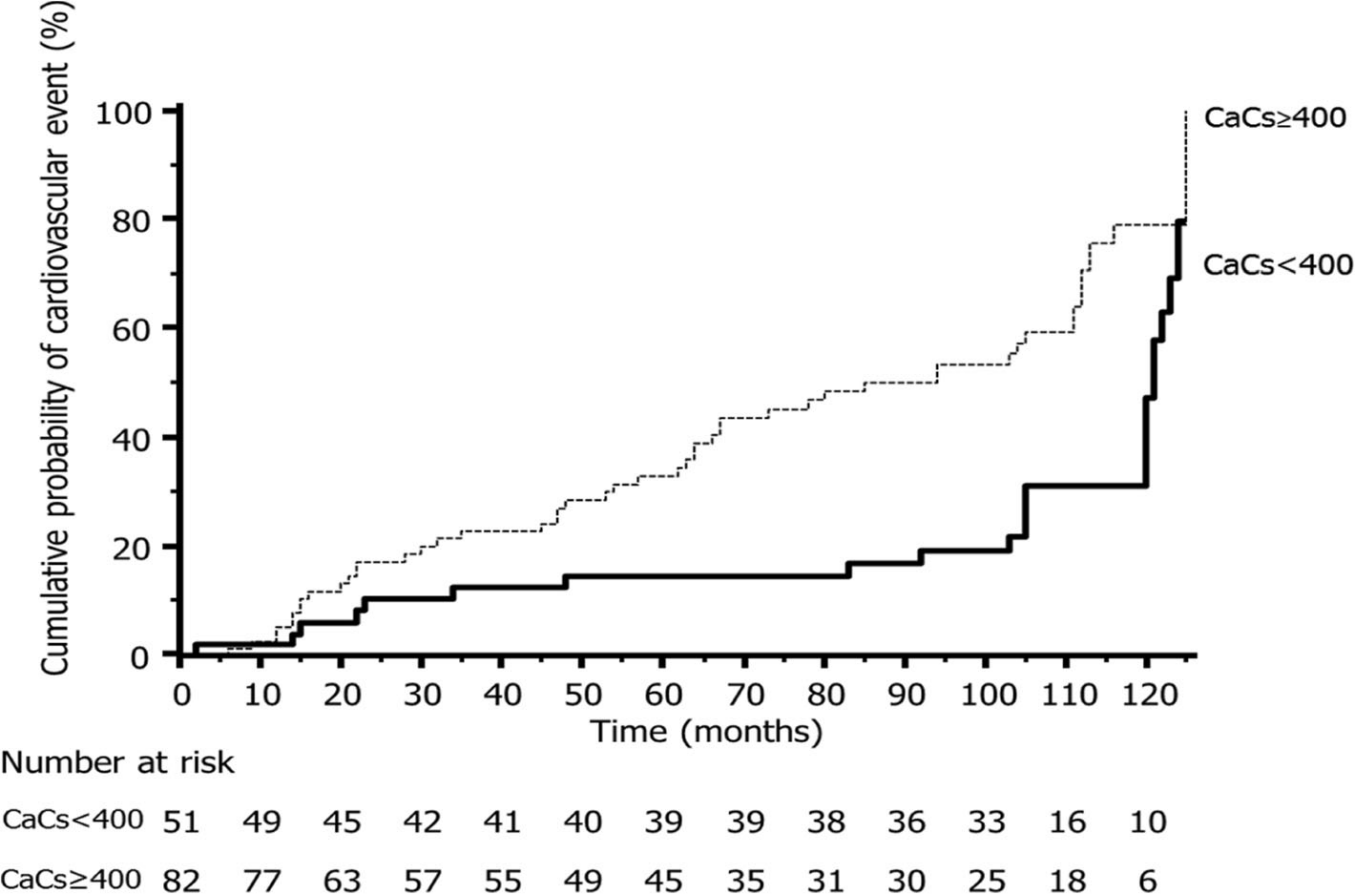
Unadjusted Kaplan Meier cumulative curve for non-fatal cardiovascular events caused by coronary arterial calcium score in Hounsfield Units (CaCs HU); group with severe coronary calcification (CaCs ≥400 HU) and group with mild-moderate coronary calcification (CaCs< 400 HU) are shown. Non-fatal cardiovascular events include heart failure, ischemic heart disease, atrial fibrillation and stroke. *p* = 0.001

A total of 292 CV events were observed during the follow-up period; 149 of them required admission to hospital. These CV events included: 82 heart failures, 17 atrial fibrillations, 29 ischemic heart diseases and 21 strokes. Figure 2 shows the unadjusted KM cumulative-curve for CV events incidence according to CaCs. The differences among these curves were statistically significant (X^2^ 10.88, *p* = 0.001).

### Coronary calcification as a predictor of total and cardiovascular mortality

On univariate Cox analysis of the sample (Table 3), the predictive variables associated with overall mortality were: CaCs≥400 HU, age and previous cardiologic events. On multivariate Cox analysis, both age and previous cardiologic events still kept a statistically significant association with total mortality-according to omnibus test (X^2^ 10.18, *p* = 0.006)- while, only in haemodialysis patients, severe CaCs seemed to have tendency on overall mortality (HR 1.55; 95%CI 0.84–2.87), although not reaching a statistical significance (Additional file 1).

**Table 3:** Univariate and multivariate Cox analysis of the factors associated with overall and cardiovascular mortality

	Overall mortality				Cardiovascular mortality			
	Univariate Cox		Multivariate Cox		Univariate Cox		Multivariate Cox	
Variable	HR (IC 95%)	p	HR (IC 95%)	p	HR (IC 95%)	p	HR (IC 95%)	p
Age	1.03 (1.00–1.05)	0.034	1.03 (1.01–1.05)	0.02	1.02 (0.98–1.07)	0.24	–	
Male	1.23 (0.72–2.10)	0.45	–		1.19 (0.57–2.50)	0.65	–	
Haemodialysis	1.2 (0.71–2.03)	0.5	–		1.49 (0.64–3.44)	0.35	–	
Haemodialysis vintage	1.0 (0.99–1.00)	0.95	–		1 (0.99–1.00)	0.38	–	
Hypertension	1.43 (0.71–2.88)	0.31	–		2.16 (0.61–7.60)	0.23	–	
Diabetes Mellitus	1.12 (0.63–2.00)	0.69	–		0.84 (0.34–2.09)	0.71	–	
Smoker	1.18 (0.57–2.43)	0.66	–		3.19 (0.91–11.2)	0.07	–	
History of stroke event	0.63 (0.32–1.24)	0.18	–		0.31 (0.11–0.87)	0.03	0.56 (0.16–1.94)	0.36
History of cardiologic event	1.63 (0.92–2.88)	0.09	1.81 (1.09–3.03)	0.02	1.3 (0.6–2.82)	0.51	–	
Chronic ischemic heart disease	1.82 (0.97–3.41)	0.06			1.58 (0.67–3.72)	0.30	–	
Antiplatelet treatment	1.15 (0.67–1.96)	0.62	–		0.91 (0.44–1.88)	0.80	–	
Statin treatment	1.43 (0.83–2.47)	0.20	–		1.36 (0.65–2.83)	0.42	–	
Vit. D treatment	1.23 (0.72–2.09)	0.44	–		1.36 (0.65–2.84)	0.41	–	
Calcium salt use	1.01 (0.59–1.74)	0.98	–		1.86 (0.86–4.01)	0.12	–	
Phosphate binder use	0.64 (0.37–1.13)	0.13	–		1.87 (0.88–4.01)	0.11	–	
Cinacalcet treatment	0.62 (0.26–1.47)	0.28	–		0.41 (0.12–1.41)	0.16	–	
Cholesterol	1.00 (0.99–1.00)	0.59	–		1.00 (0.99–1.01)	0.77	–	
LDL-cholesterol	0.99 (0.99–1.00)	0.20	–		1.00 (0.99–1.01)	0.65	–	
HDL-cholesterol	1.01 (0.99–1.01)	0.50	–		1.00 (0.99–1.00)	0.82	–	
Triglycerides	0.99 (0.995–1)	0.02			1.00 (0.73–1.30)	0.08		
Uric acid	0.95 (0.82–1.11)	0.50	–		0.97 (0.73–1.30)	0.83	–	
HbA1c	1.21 (0.94–1.53)	0.14	–		1.21 (0.83–1.77)	0.32	–	
Albumin	1.06 (0.58–1.93)	0.86	–		0.40 (0.12–1.35)	0.14	–	
Creatinine	0.99 (0.93–1.05)	0.64	–		0.92 (0.84–1.01)	0.08	–	
Urea	1.00 (0.99–1.01)	0.32	–		1.00 (0.99–1.00)	0.24	–	
Phosphorous	0.90 (0.76–1.06)	0.19	–		1.06 (0.79–1.41)	0.72	–	
Calcium	0.93 (0.68–1.30)	0.68	–		0.39 (0.19–0.84)	0.02	0.37 (0.18–0.78)	0.01
Ca X P product	0.99 (0.97–1.00)	0.16	–		1.00 (0.12–1.35)	0.14	–	
Bicarbonate	1.03 (0.97–1.09)	0.4	–		1.11 (1–1.23)	0.06		
Intact PTH	1.00 (0.99–1.00)	0.94	–		1.00 (0.99–1.00)	0.30	–	
TSH	1.07 (1.01–1.14)	0.03			1.05 (0.98–1.12)	0.15	–	
CRP	1.00 (0.99–1.007)	0.76	–		1.02 (0.98–1.07)	0.26	–	
CaCs ≥400 HU	2.15 (0.95–4.84)	0.07	1.55 (0.84–2.87)	0.16	5.91 (1.18–29.48)	0.03	5.01 (1.28–19.59)	0.02

The same strategy was carried out to assess the role of the CaCs as a predictor of CV mortality. On the univariate Cox analysis, those variables associated with CV mortality were: CaCs ≥400 HU, previous stroke and serum calcium. On multivariate Cox analysis, CaCs≥400 HU and serum calcium -according to omnibus test (X^2^ 14.47, *p* = 0.002)- still appeared to have an influence on CV mortality (Table 3).

Finally, in a separate analysis that considered CaCs as a continuous variable, we found that a doubling of the calcium score increased the estimated probability of both overall (HR 1.19; 95%CI 1.1–1. 29) and CV (HR 1.43; 95%CI 1.19–1.72) mortality by 19 and 43% respectively, during a median follow-up period of 87.5 (29.5–111) months.

## Discussion

In recent years, mortality of patients in haemodialysis has decreased due to improvements in technique. According to the United States Renal Data System, life expectancy of patients once they start haemodialysis ranges between 8 and 4.5 years. Increasing length of time on dialysis is a related to higher mortality rates [19].

In the present study, we explore the predictive value of CaCs for total and CV mortality in advanced CKD and haemodialysis population. Our results point out that CaCs≥400 HU could be a predictor of higher CV mortality in this population, on a long-term basis. Unfortunately, we were not able to identify a clear relationship between CaCs and overall mortality in the whole sample. A possible limitation of the study might be the use of a relatively small sample size; therefore, further studies should be designed to explore this hypothesis.

In addition, we found that a doubling of the CaC score increased the estimated probability of CV mortality by approximately 43% in a 10-year period. In the Detrano study, subjects aged 45 to 84 years with no clinical cardiovascular disease before enrolment were followed for a median of 3.8 years; authors found that a doubling of calcium score resulted in a 20% increase in the risk of a major coronary event (myocardial infarction or death from coronary heart disease) [18]. Our findings support previous studies that examined the relationship between CaC measured by MSCT and mortality in advanced CKD patients. These studies were performed mostly in advanced CKD patients and haemodialysis patients separately [10, 20–22], whereas we have preferred to include both types of patients together in our analysis. Moreover, to our knowledge, present data include the longest follow-up period in these patients until now.

In CKD patients, Watanabi et al. investigated a heterogeneous sample of 117 non dialyzed patients with CKD stages 2 to 5. After a follow-up period of 24 months, they found that the presence of CaCs ≥400 HU was associated with a shorter cardiovascular and hospitalization event-free time and a lower survival rate [20]. Recently, Chen et al. have addressed, in larger CKD cohort with a long follow-up, the association between CaC measured by MSCT and the risk of CV disease and mortality [23]. They found that CaCs is related to risk of CV diseases and improves risk predicted models. However, there are some notably differences with our current research: Chen et al. included only 20% of cases with an estimated glomerular filtration < 30 ml/min, specifically excluded haemodialysis patients, and only considered overall mortality. Although their main conclusions are completely valid, their results did not provide information about the prognostic value of CaC concerning CV mortality in advanced CKD, and it is in this aspect in which our results stress the value of this measurement in improving prognostic predictions.

Shantouf et al. found that haemodialysis patients with CaCs 0 HU had a higher rate of 6-year event-free survival (89%) compared to those with a CaCs> 400 HU (58%). After adjustment for standard CV risk factors and bone-mineral metabolism parameters, they reported a higher total mortality in CaCs> 400 HU group [2].

Some authors have suggested that CaCs could be an independent predictor of all-cause mortality in haemodialysis. After adjusting for other CV risk factors, Matsuoka et al. found that the 5-year cumulative survival rate was significantly different between patients with low CaCs (84%) compared to those with high CaCs (68%), although with an adjusted relative risk of death of 1.001 [10]. Block et al. (2007) described that baseline CaCs> 400 HU was a significant predictor of all-cause mortality (HR 4.5, 95%CI 1.33–15.14) in 127 incident haemodialysis patients during a 4-year follow-up period [22].

In a 7-year follow-up study, reported by Shimoyama [6], which was carried out in 200 haemodialysis patients, 51% of deaths were related to CV events. Much lower CV mortality was found in the lowest CaCs and similar results were reported in all-cause mortality. In a prospective study of a similar sample size, Noce et al. also found a significant difference in CV mortality in patients with CaCs≤400, using Kaplan-Meier survival analysis, compared to those in the group of CaCs>400HU; 49.7% died due to a major CV event during the follow-up period of 7 years [24].

In our study, we did not include CV disease subtypes in Cox analysis. However, it would have been interesting to analyse the subtypes of CV mortality in the haemodialysis group according to their CaCs, since non-atheromatous CV events could predominate in these patients [14]. Matsushita et al. just assessed CV disease subtype. The prediction model with CaC was superior to intima media thickness and ankle-brachial index, only for coronary heart disease and heart failure prediction, regardless of CKD status [25].

As reported by other authors, we found a high prevalence of CaC, ranging 0–8798 HU. This asymmetric distribution is consistent with other similar studies [6, 16, 26]. Moreover, patients with high CaCs had worse renal function than those with low CaCs. Also, there were more haemodialysis patients in the group of severe calcifications. This finding supports the inverse relationship between glomerular filtration rate and CaC [1, 7]. As reported in previous studies, we found that patients with high CaCs were older, with higher prevalence of previous cardiologic events and type 2 diabetes [2, 8, 10, 27]. It is well known that diabetes is a risk factor for developing atherosclerosis and vascular calcification [28, 29].

Our data showed that patients with high CaCs had lower albumin levels than patients with mild or moderate CaC. Malnutrition-inflammation complex syndrome is a common phenomenon in dialysis patients, and is related to increased morbidity and mortality. In dialysis patients, there seems to be an association between malnutrition-inflammation complex syndrome and vascular calcification [30, 31]. Therefore, lower albumin level, as a marker of malnutrition and inflammation, could be a risk marker of CaC. This finding, however, needs to be validated conducting larger sample prospective studies.

We did not find a significant association between CaC and any biochemical parameter related to mineral metabolism or other traditional CV risk parameters. This lack of association could be explained by the small sample size, the study design or the clinical characteristics of our cohort. It should be mentioned that biochemical parameters were measured only once at baseline, and a single baseline laboratory value may not reflect the time-averaged exposure.

Traditionally, hypercalcemia, hyperphosphatemia and secondary hyperparathyroidism have been associated with vascular calcification in patients with CKD. However, it has been described that excessive suppression of PTH, or relative hypoparathyroidism, leads to a decrease in bone remodelling or adynamic bone. It is also related to a greater progression of vascular and coronary calcification. Therefore, calcium and PTH levels do not always are correlated with net calcium balance and vascular calcification severity [32].

Therapeutic changes were not recorded, so might be they have had some influence on endpoints. In relation to this point, it is known that CKD and dialysis patients treated with non-calcium-based phosphate binders have a reduction in all-cause mortality compared with those that use calcium-based phosphate binders [33, 34]. Besides, it has been reported that a low dialysate calcium level could reduce progression rate of CaC in haemodialysis patients [35]. Besides, the treatment provided to patients was aimed to control disturbances of mineral metabolism adequately, and this pharmacological intervention could have conditioned the results obtained. Another potential bias might be related to the design of the enrolment if it considered that those patients of incident haemodialysis and severe CaC might experience a premature death (< 6 months). Though, we set a minimum period of 6 months of permanence in the hemodialysis program to minimize this theoretical bias. Thus, those cases incident in hemodialysis, whose death in a period inferior to 6 months, were excluded. All enrolled cases were previously followed up in the Nephrologist outpatient office. And, in fact, the ANSWER study reports that the most potent predictor of early mortality was inadequate pre-dialysis treatment [36].

Moreover, CaC in our cohort was only measured just once, at baseline conditions. Therefore, we couldn’t report number of patients with CaCs < 400 HU who have change to CaCs > 400 HU during the follow up.

## Conclusions

We conclude that, within the limits of our relatively small sample size, CaC is highly prevalent in advanced CKD patients. Age, diabetes and haemodialysis therapy may be associated with CaC severity. CaCs seems to be a good long-term predictor of CV mortality in patients with advanced CKD, but larger prospective studies are needed to confirm the clinical utility of performing routine MSCT in this population in order to assess CV risk.

## Additional file

Additional file 1: **Tables S1 and S2.** Description of cardiovascular events and mortality. Multivariate proportional Cox analysis in the haemodialysis group. (DOCX 16 kb)

## Abbreviations

CaC: Coronary calcification
CaCs: Coronary calcification score
CI: Confidence interval
CKD: Chronic kidney disease
CV: Cardiovascular
HD: Haemodialysis
HR: Hazard ratio
HU: Hounsfield Units
IQR: Interquartile range
KM: Kaplan-Meier
MSCT: Multi-layer spiral computed tomography
OR: Odds ratio
SD: Standard deviation
