# Supplementary material for: Coronary calcification as a predictor of cardiovascular mortality in advanced chronic kidney disease: a prospective long-term follow-up study
Source: BMC Nephrol. 2019 May 28;20:188. doi: 10.1186/s12882-019-1367-1 (PMC6537175; doi:10.1186/s12882-019-1367-1)
Supplement: Supplementary file 1 — Tables S1 and S2. Description of cardiovascular events and mortality. Multivariate proportional Cox analysis in the haemodialysis group. (DOCX 16 kb) [file 12882_2019_1367_MOESM1_ESM.docx]

Additional file 1

**Table S1.** Cardiovascular events and mortality

|  | N (%)/ Median (IQR)/Mean (SD) | Total cumulative events (n) |
| --- | --- | --- |
| Mean survival (months) | 35 (15-68) |  |
| Previous cardiovascular event  Heart failure  Atrial fibrillation  Ischemic cardiac disease  Stroke  Cardiovascular hospitalization |  | 292  82  17  29  21  149 |
| Cardiovascular mortality (%) | 32 (23.2) |  |
| Non- Cardiovascular mortality (%) | 37 (26.8) |  |
| Unknown cause of death (%) | 11 (8) |  |
| Overall mortality (%) | 80 (58) |  |

Data are presented as number of patients and percentage [ N (%)], mean and standard deviation (± SD).

**Table S2**. Multivariate proportional Cox analysis in the hemodialysis group

1. Overall mortality. Hemodialysis.

| Covariates | HR | 95%CI | p |
| --- | --- | --- | --- |
| Age | 1.05 | 1.02-1.09 | 0.002 |
| Cardiologic event | 1.58 | 0.86-2.89 | 0.14 |
| CaC≥400 HU | 4.12 | 1.83-9.30 | 0.001 |

Cardiologic event: previous cardiologic events (ischemic heart disease, heart failure); CaC score (HU): coronary arterial calcification score (Hounsfield Units);

1. Cardiovascular mortality. Hemodialysis.

| Covariates | HR | 95%CI | p |
| --- | --- | --- | --- |
| Serum Calcium | 0.39 | 0.16-0.98 | 0.04 |
| CaCS≥400 HU | 3.02 | 0.43-21.3 | 0.27 |

CaC score (HU): coronary arterial calcification score (Hounsfield Units);
